# Association between habitual sleep duration and Alzheimer’s disease: A cross-sectional study based on NHANES 2007–2018

**DOI:** 10.1097/MD.0000000000050086

**Published:** 2026-08-14

**Authors:** Guang Yang, Wenzhe Hou, Jiawen Wu, Xian Yang, Ye Yang, Qiqi Chen, Yanli Liu

**Affiliations:** a Department of Neurology, Kunshan Hospital of Traditional Chinese Medicine, Suzhou, China; b Department of Neurology, Northern Jiangsu People’s Hospital, Yangzhou, Jiangsu Province, China; c Department of Pharmacy, Nanjing Drum Tower Hospital, Affiliated Hospital of Medical School, Nanjing University, Nanjing, Jiangsu Province, China; d Department of Radiology, Kunshan Traditional Chinese Medicine Hospital, Kunshan, China.

**Keywords:** All-cause mortality, Alzheimer’s disease, National Health and Nutrition Examination Survey, Public Health, Sleep time

## Abstract

Given the potential importance of sleep disturbances in the pathophysiology of Alzheimer’s disease (AD), as well as the fact that habitual sleep duration – an observable and modifiable lifestyle factor – has become a significant potential target for early risk warning of neurodegenerative diseases, this study utilizes data from the National Health and Nutrition Examination Survey to thoroughly examine the association between AD and habitual sleep patterns among adults in the United States. It aims to determine the correlations among sleep duration, Alzheimer’s disease, and all-cause mortality, thereby providing population-based evidence for the early prevention and public health intervention of AD. Using a cross-sectional design, 29,692 participants were selected. A multiple logistic regression model was used to assess the association between sleep duration and Alzheimer’s disease. Additionally, all-cause mortality status was derived from matched records obtained from the United States National Death Index. The Cox regression model was used to calculate the hazard ratios and 95% confidence intervals of all-cause mortality associated with the relationship between sleep duration and AD. Our analysis revealed a significant positive correlation between increased sleep time and AD prevalence, a relationship that persisted in the adjusted model. Subgroup analysis showed that this association remained consistent across different populations. This study clarifies the effectiveness of sleep time as a predictive tool for AD and underscores its importance in AD assessment. Based on the predictive value of sleep time, our study advocates incorporating body composition factors into AD risk assessment, thus expanding the scope of Alzheimer’s disease prevention strategies.

## 1. Introduction

Alzheimer’s disease (AD) is the leading cause of dementia and is implicated in an estimated 60% to 80% of cases. Current global estimates indicate that approximately 50 million patients are afflicted by this debilitating neurodegenerative condition, which imposes profound life alterations on tens of millions of caregivers who must confront the progressive nature of their family members’ cognitive decline.^[1]^ According to recent estimates, the number of dementia cases in Europe is set to double by 2050, while global figures may triple. Notably, applying a biological definition of Alzheimer’s reveals a potential threefold increase in these projections, hinting at widespread under-diagnosis.^[2]^ Dominant mutations in the amyloid precursor protein, presenilin 1 and presenilin 2 genes account for a small fraction of all AD cases. Symptom presentation in these genetic cases typically begins before 65 years of age, a defining feature of the early onset form.^[3,4]^ The majority of Alzheimer’s cases are late-onset AD, a sporadic form that emerges in older adults. Although not directly inherited, its development is strongly influenced by polygenic risk architecture, with the E4 allele in the apolipoprotein E gene – a variant carried by approximately 16% of the population, which is the most significant genetic susceptibility factor.^[5]^ Certain lifestyle behaviors and health conditions are established risk factors for this disease. Inadequate nutrition and sedentary patterns, combined with comorbidities such as diabetes, cerebrovascular disease, head injuries, and persistent stress, are broadly linked to a heightened risk profile.^[4,6,7]^

Sleep is one of the most highly conserved features in the animal kingdom, indicating a strong evolutionary requirement.^[8]^ Human sleep can be characterized by dimensions such as duration, timing, efficiency, and regularity, and each dimension is sometimes associated with adverse health outcomes.^[9-11]^ However, sleep duration has been extensively studied and is associated with outcomes such as obesity, cardiovascular disease, and mortality. Both abnormally long and abnormally short sleep durations are associated with multiple mental disorders, including major depressive disorder, anxiety, and psychosis, although a causal relationship between sleep duration and these diseases has not been determined.^[12-16]^

As a potential biomarker for AD pathology, sleep offers the possibility of noninvasive monitoring. This facilitates both the assessment of future disease risk and tracking of interventional responses in clinical trials.^[17-20]^ There is a pressing need to clarify the dynamic interplay between sleep and AD, both of which are modulated by aging. Future research must map the concurrent changes in sleep and cognition that occur with advancing age and across the continuum of Alzheimer’s pathology.^[21-25]^ In previous research, the primary sources of data on sleep duration and related parameters were self-reported measures. In addition, measurements of sleep quality, such as sleep efficiency and non-rapid-eye-movement, slow-wave activity, are related to AD pathology and cognitive function.^[26-31]^ The potential link between sleep duration and AD prevalence remains unexplored in scientific literature. Therefore, this study attempted to use data from the National Health and Nutrition Examination Survey (NHANES) in the United States from to 2007–2018 to clarify the potential correlation between sleep time and AD in a representative sample of the US population.

## 2. Materials and methods

### 2.1. Study population

This study utilized data from the 2007–2018 cycles of the National Health and Nutrition Examination Survey, a comprehensive cross-sectional research program that evaluates the health and nutritional status of both adult and pediatric populations in the United States through a combination of standardized interviews and physical examinations. We selected these cycles for several reasons. First, these cycles consistently included the key variables necessary for our analysis, such as self-reported sleep duration, AD diagnostic information, and a comprehensive set of covariates (e.g., demographic, lifestyle, and comorbidity data). Second, the linkage of NHANES data with the National Death Index (NDI) through 2019 allowed us to assess all-cause mortality with sufficient follow-up time. Data from 2019 onward were not fully available or linked to mortality outcomes at the time of our analysis, as NHANES data release and linkage processes typically lag by several years. We acknowledge that this study is a secondary analysis of publicly available NHANES data. All data were collected by the National Center for Health Statistics using standardized protocols, and we did not conduct primary data collection. This approach allows for the use of a large, nationally representative sample, enhancing the generalizability of our findings. The interview component collected detailed information on the demographic background, socioeconomic status, dietary intake, and various health-related topics.

**Figure 1.**
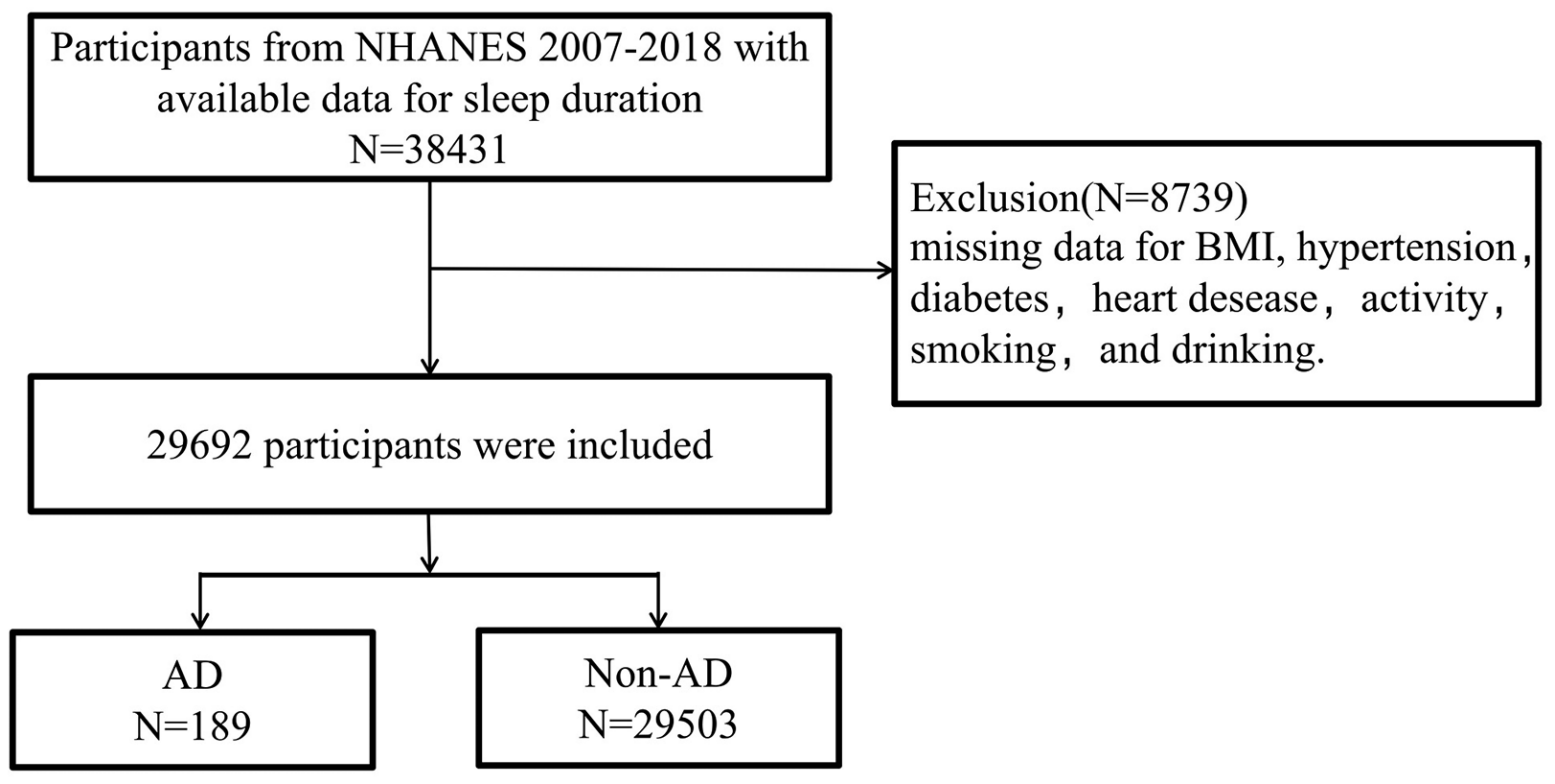
Flowchart of participants selection. AD = Alzheimer’s disease, BMI = body mass index, NHANES = National Health and Nutrition Examination Survey.

The research protocol was approved by the Institutional Review Board of the National Center for Health Statistics and the Centers for Disease Control and Prevention, and written informed consent was obtained from all participants. From the initial sample of 38,431 individuals, we applied the following exclusion criteria: age < 20 years (this cutoff aligns with the typical onset of early adulthood and is consistent with other epidemiological studies of adult-onset conditions using NHANES), incomplete data for depression diagnosis or Non-High-Density Lipoprotein Cholesterol to High-Density Lipoprotein Cholesterol Ratio derivation (depression is a known confounder in sleep and AD research; incomplete data on depression or related indices would limit our ability to adjust for this important variable, potentially biasing the results), and missing values for any of the key covariates (alcohol consumption, BMI, educational attainment, PIR, dietary intake, hypertension, diabetes, and smoking status). The participant selection procedure is illustrated in Figure 1.

### 2.2. Sleep duration

During the 7 cycles of NHANES from 2007 to 2018, sleep duration was assessed by asking, “How much sleep do you usually get at night on weekdays or workdays?” The participants were asked to indicate the usual number of hours of sleep per day. Sleep duration was divided into 3 commonly cited 3,4 categories: short, <7 hours; normal, 7 to 8 hours; and long, ≥8 hours of sleep. An alternate analysis of short sleep, defined as <6 hours (another definition commonly cited in the literature), was also conducted.

### 2.3. Ascertainment of AD

AD case status was assigned to individuals based on either self-reported diagnosis or pharmacotherapy with Rivastigmine, Galantamine, Donepezil, or Memantine. This definition was supplemented by mortality data from the NDI, where an AD diagnosis on the death certificate qualified a participant as a case.

### 2.4. Outcome ascertainment

This study defined all-cause mortality as the primary endpoint, referring to death from any cause that occurred within the follow-up period. All-cause mortality was included as a composite endpoint to capture the overall survival impact associated with sleep duration in individuals with AD. Since AD is a progressive neurodegenerative disorder often accompanied by multiple comorbidities, cause-specific mortality can be difficult to ascertain accurately. All-cause mortality provides a robust and clinically meaningful measure of overall health impact. In our study, we used mortality data from the NDI to identify deaths through December 31, 2019. This allowed us to examine whether sleep duration was associated with increased mortality risk in AD patients, independent of specific causes of death. Using respondent sequence numbers, individuals with AD were monitored through December 31, 2019, to determine survival status and compute follow-up duration (Centers for Disease Control and Prevention, 2022e). The observation period for each participant was quantified in person-months, commencing from the baseline mobile examination date and concluding either at the date of death or the end date (December 31, 2019), whichever came earlier.

### 2.5. Covariates

We incorporated a comprehensive set of covariates, including age, sex, race, education, marital status, smoking status, alcohol consumption, hypertension, diabetes, angina pectoris, arthritis, heart disease, vigorous activity, and sleep duration. The covariates included in our models were selected based on established evidence from the literature regarding potential confounders or effect modifiers in the relationship between sleep and AD – Demographic factors: age, sex, and race are fundamental determinants of both sleep patterns and AD risk. Socioeconomic factors: education and poverty-income ratio (PIR) are associated with access to healthcare, health behaviors, and AD prevalence. Lifestyle factors: smoking and alcohol consumption are linked to both sleep disturbances and neurodegenerative risk. Comorbidities: hypertension, diabetes, heart disease, and vigorous physical activity are known to influence both sleep quality and AD pathogenesis. Body composition: BMI was included as a marker of metabolic health, which may mediate or confound the sleep-AD relationship. Adjustment for these covariates aimed to isolate the independent association between sleep duration and AD. Education level was dichotomized, with 1 group defined as having completed education beyond high school. Smoking status included having smoked at least 100 cigarettes in their lifetime. Alcohol consumption was defined as the consumption of at least 12 alcoholic beverages.

### 2.6. Statistical analysis

We conducted a comprehensive assessment of participant demographics stratified by AD status. Between-group comparisons for categorical and continuous variables were performed using weighted chi-square tests and *t*-tests, respectively. The association between sleep duration and AD diagnosis was evaluated using multivariable logistic regression, whereas its relationship with AD severity was assessed using multivariable linear regression. To characterize the potential nonlinear patterns, we implemented a weighted generalized additive model with smooth curve fitting. Furthermore, we performed stratification and interaction tests to examine the consistency of the sleep-AD relationship across different population subgroups.

We considered p-values below 0.05 in 2-tailed tests to be statistically significant. All analytical procedures were implemented using R Statistical Software (Version4.2.2, available at http://www.R-project.org, developed by The R Foundation) and the Free Statistics analysis platform (Version 1.8, Beijing, China).

## 3. Results

This study included a total of 29,692 participants. The cohort was divided into 2 groups: 29,503 individuals without AD (accounting for 99.36%) and 189 individuals diagnosed with AD (accounting for 0.64%). As shown in Table 1, the overall weighted prevalence of AD was 0.64% (Table 1). Comparative analysis between these groups revealed that several factors such as sex, age, race, education, marital status, PIR, BMI, drinking history, heart disease, hypertension, and vigorous exercise were associated with AD status. A key observation from Table 1 pertains to sleep duration, which was significantly prolonged in the AD group (*P* < .001), suggesting its potential relevance to the disease. In contrast, the body mass index (BMI) did not differ significantly between the groups (*P* = .129).

**Table 1:** Characteristics of participants in NHANES 2007–2018.

Variable	Total N = 29692	Non-AD N = 29503	AD N = 189	*P*
Sex, n (%)				.001
Male	14588 (48.7)	14506 (48.8)	82 (34.6)	
Female	15104 (51.3)	14997 (51.2)	107 (65.4)	
Age, yr, n (%)				<.001
20–59	19016 (71.1)	19010 (71.4)	6 (5.7)	
≥60	10676 (28.9)	10493 (28.6)	183 (94.3)	
Race, n (%)				.021
Mexican American	4450 (8.4)	4439 (8.5)	11 (2.6)	
Other Hispanic	3102 (5.8)	3075 (5.8)	27 (6.9)	
Non-Hispanic White	12344 (67.0)	12243 (67.0)	101 (74.3)	
Non-Hispanic Black	6342 (11.0)	6304 (11.0)	38 (10.0)	
Other Race	3454 (7.7)	3442 (7.7)	12 (6.2)	
Education level, n (%)				.007
<high school	7121 (15.2)	7056 (15.3)	65 (26.3)	
high school	6818 (23.1)	6783 (23.1)	35 (19.3)	
>high school	15753 (61.5)	15664 (61.5)	89 (54.4)	
Married, n (%)				.056
No	14555 (45.1)	14478 (45.1)	77 (36.2)	
Yes	15137 (54.9)	15025 (54.9)	112 (63.8)	
PIR, n (%)				<.001
<1.3	8405 (19.8)	8349 (19.9)	56 (15.3)	
1.3–3.5	9757 (40.3)	9683 (40.2)	74 (62.7)	
>3.5	11530 (39.8)	11471 (39.9)	59 (22.0)	
BMI, kg/m^2^, n (%)				.129
<25	8405 (29.1)	8349 (29.1)	56 (28.4)	
25 to <30	9757 (32.9)	9683 (32.9)	74 (41.2)	
≥30	11530 (38.0)	11471 (38.0)	59 (30.4)	
Alcohol drink, n (%)				<.001
No	12677 (35.6)	12534 (35.4)	143 (71.8)	
Yes	17015 (64.4)	1696 (64.6)	46 (28.2)	
Smoking, n (%)				.785
No	16476 (55.5)	16373 (55.5)	103 (56.7)	
Yes	13216 (44.5)	13130 (44.5)	86 (43.3)	
Heart disease, n (%)				<.001
No	27163 (93.2)	27027 (93.3)	136 (71.6)	
Yes	2529 (6.8)	2476 (6.7)	53 (28.4)	
Diabetes, n (%)				<.001
No	25706 (90.1)	25568 (90.1)	138 (76.1)	
Yes	3986 (9.9)	3935 (9.9)	51 (23.9)	
Hypertension, n (%)				<.001
No	10914 (32.4)	10792 (32.2)	122 (64.0)	
Yes	18778 (67.6)	18711 (67.8)	67 (36.0)	
Vigorous activity, n (%)				<.001
No	23,199 (73.9)	23,014 (73.8)	185 (96.4)	
Yes	6493 (26.1)	6489 (26.2)	4 (3.6)	
Sleep duration, n (%)				<.001
<7	10197 (31.4)	10161 (31.4)	36 (12.5)	
7–8	15005 (53.8)	14929 (53.8)	76 (43.4)	
>8	4490 (14.9)	4413 (14.7)	77 (44.2)	

Data are displayed as unweighted frequency counts (weighted percentage). The Rao-Scott chi-square test for categorical variables was used in this analysis.

AD = Alzheimer’s disease, BMI = body mass index, NHANES = National Health and Nutrition Examination Survey.

The Cox regression analysis showed that the prevalence of AD was significantly associated with a significant increase in sleep duration (Table 2). After adjusting for covariates, 2 models were developed. Model 1 was adjusted for demographic factors, including age, sex, and race. After adjusting for age, sex, and race factors and adjusting for covariates (odds ratio [OR] 1.53, 95% confidence interval [CI]: 1.35–1.74, *P* < .001), there was a significant association between sleep duration and the risk of AD. Model 2 was further adjusted for educational level, marital status, PIR, BMI, smoking, alcohol consumption, hypertension, heart disease, diabetes mellitus, and vigorous exercise. After the most comprehensively adjusted classification model (OR 1.48, 95% CI: 1.32–1.66, *P* < .001), it was found that compared with <7 hours of sleep time, the risk of AD for 7 to 8 hour of sleep time would increase by 93% (OR: 1.93, 95% CI: 1.19–3.15). Compared with <7 hours of sleep time, the prevalence of AD for more than 8-hour sleep time would increase by 197% (OR: 4.97, 95% CI: 2.79–8.88).

**Table 2:** Association between sleep duration and AD.

		Unadjusted		Model 1		Model 2	
		OR (95% CI)	*P*	OR (95% CI)	*P*	OR (95% CI)	*P*
Sleep duration/d (h)	Continues	1.65 (1.49, 1.84)	<.001	1.53 (1.35, 1.74)	<.001	1.48 (1.32, 1.66)	<.001
	<7	Ref		Ref		Ref	
	7–8	2.03 (1.26, 3.28)	.004	1.81 (1.12, 2.92)	.015	1.93 (1.19, 3.15)	.009
	>8	7.54 (4.27, 13.32)	<.001	5.90 (2.86, 9.07)	<.001	4.97 (2.79, 8.88)	<.001
	*P* for trend		<.001		<.001		<.001

Model 1 was adjusted for age, sex, and race.

Model 2 was adjusted for age, sex, race, education level, marital status, PIR, BMI, alcohol consumption, smoking, hypertension, heart disease, diabetes, and vigorous activity.

AD = Alzheimer’s disease, BMI = body mass index, CI = confidence interval, OR = odds ratio, PIR = poverty-income ratio.

We employed multivariate Cox regression analyses using 2 distinct statistical models to investigate the relationship between sleep duration and all-cause mortality. The prevalence of AD was significantly associated with a significant increase in sleep-related mortality (Table 3). We constructed 2 multivariate models to assess the robustness of our findings. Model 1 was adjusted for the basic demographic characteristics (age, sex, and race). After these adjustments, no significant association was observed between AD prevalence and sleep-related mortality in the continuous model (OR 1.17, 95% CI: 1.02–1.34, *P* = .515). Model 2 extended these adjustments to include socioeconomic, lifestyle, and comorbidity factors such as education, marital status, PIR, BMI, smoking, alcohol use, hypertension, heart disease, diabetes, and vigorous exercise. After the most comprehensively adjusted classification model (OR 1.17, 95% CI: 1.01–1.35, *P* = .449), it was found that compared with <7 hours sleep time, for >8 h sleep time, the mortality rate of AD increased by 7% (OR: 1.07, 95% CI: 0.50–2.29, *P* = .869).

**Table 3:** Association between sleep duration and mortality in AD participants.

		Unadjusted		Model 1		Model 2	
		HR (95% CI)	*P*	HR (95% CI)	*P*	HR (95% CI)	*P*
Sleep duration/d (h)	Continues	1.18 (1.03, 1.36)	.019	1.17 (1.02, 1.34)	.025	1.17 (1.01, 1.35)	.040
	<7	Reference		Reference		Reference	
	7–8	0.56 (0.31, 1.01)	.054	0.54 (0.29, 1.01)	.055	0.59 (0.30, 1.17)	.131
	>8	0.91 (0.50, 1.66)	.755	0.90 (0.47, 1.75)	.758	1.07 (0.50, 2.29)	.869
	*P* for trend		.534		.515		.449

Model 1 was adjusted for age, sex, and race.

Model 2 was adjusted for age, sex, race, education level, marital status, PIR, BMI, alcohol consumption, smoking, hypertension, heart disease, diabetes, and vigorous activity.

AD = Alzheimer’s disease, BMI = body mass index, HR = hazard rate, PIR = poverty-income ratio.

**Figure 2.**
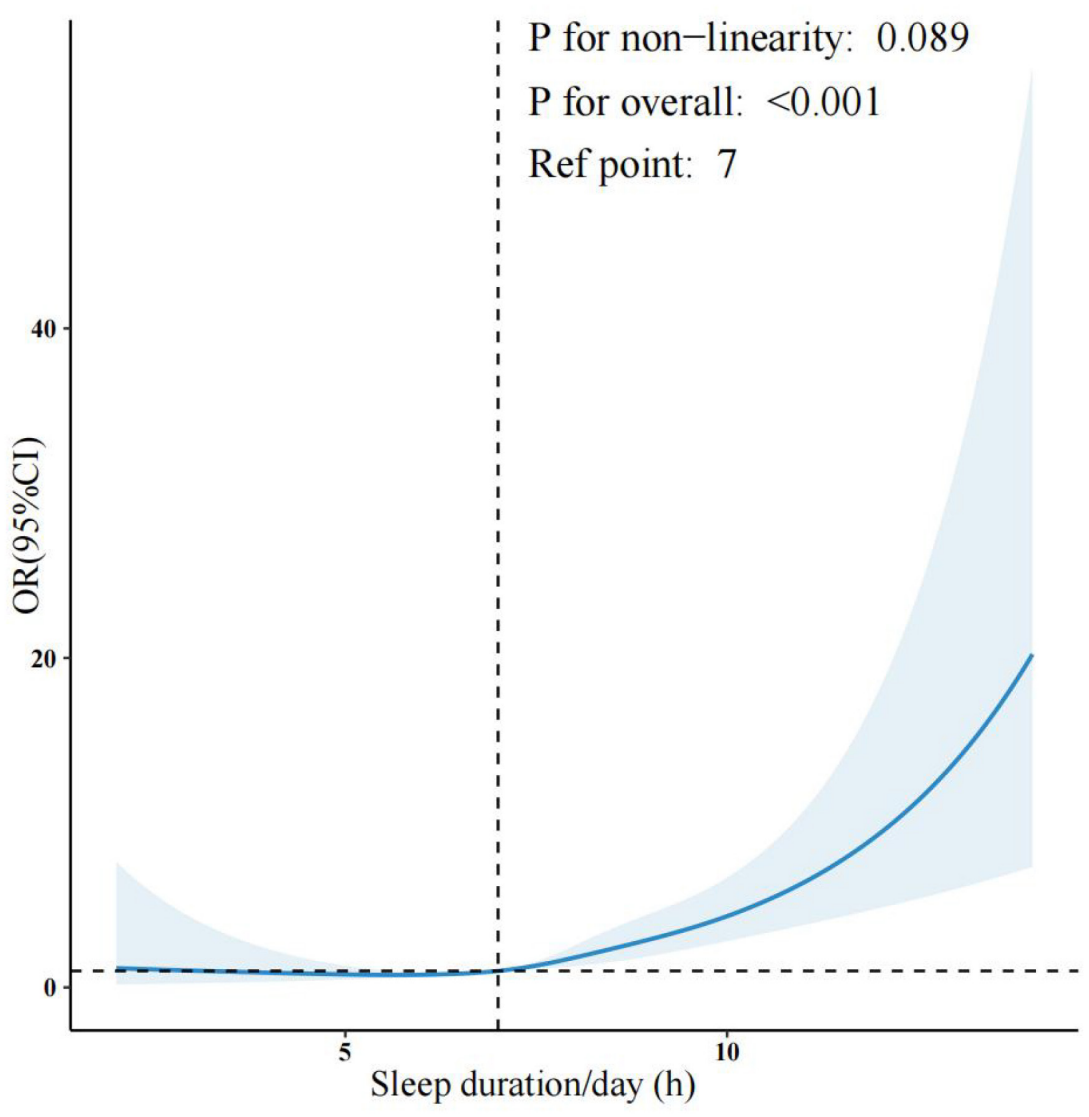
The association between sleep duration and Alzheimer’s disease. CI = confidence interval, OR = odds ratio.

The restricted cubic spline model, as depicted in Figure 2, revealed a monotonically increasing relationship between sleep duration and incidence rates. This analysis substantiated a significant linear trend, indicating that extended sleep duration is positively associated with higher mortality among patients with AD.

**Figure 3.**
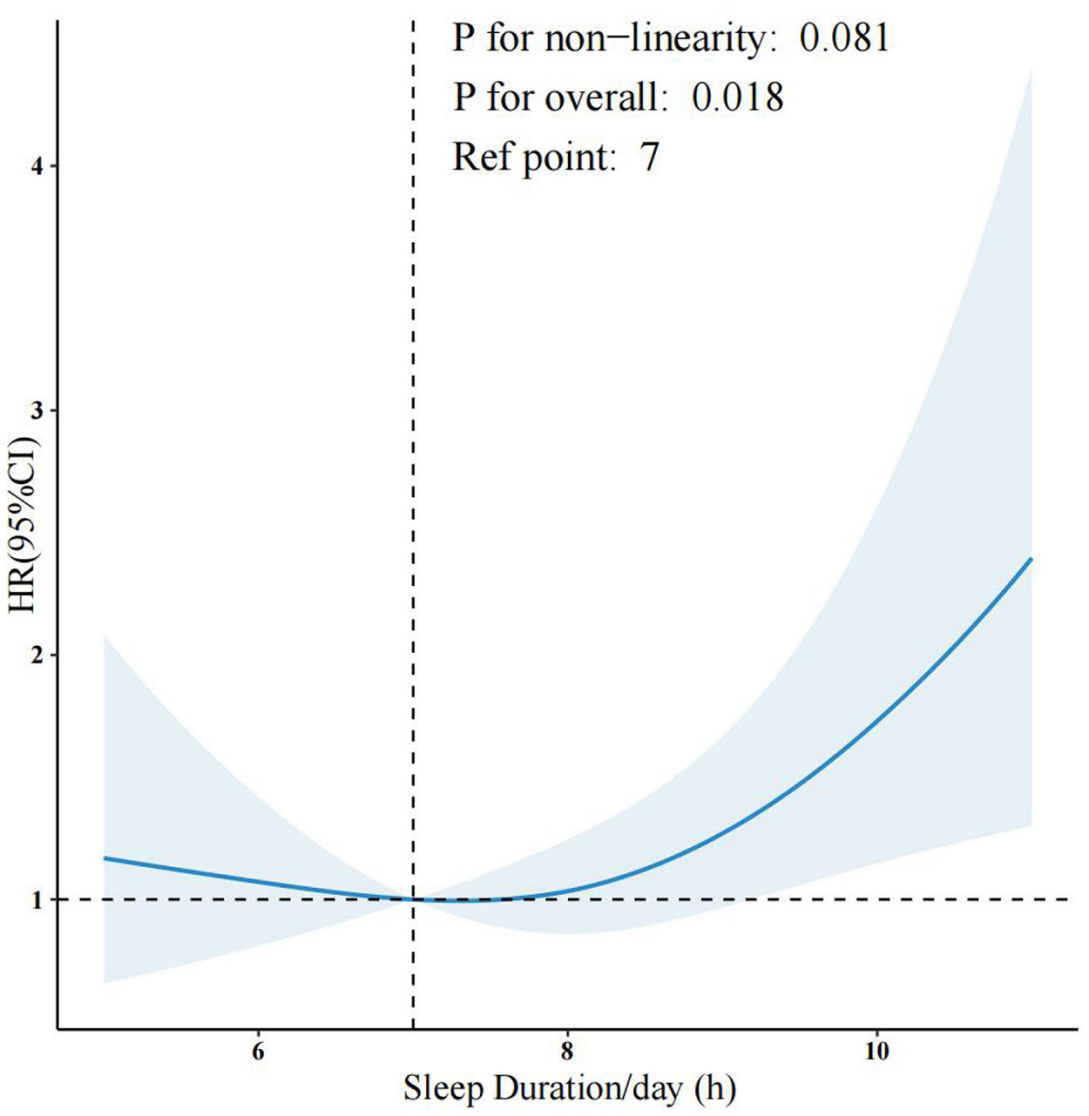
The restricted cubic spline (RCS) plot of the association between sleep duration and the mortality rate of Alzheimer’s disease. CI = confidence interval, HR = Hazard ratio. RCS = restricted cubic spline.

We used a generalized model that included smooth curve fitting to verify the nonlinear association between sleep duration and AD. When the sleep duration was <7 hours, the change in death risk with age was not very obvious; when the sleep duration was more than 7 hours, the death risk increased as age increased (Fig. 3).

**Figure 4.**
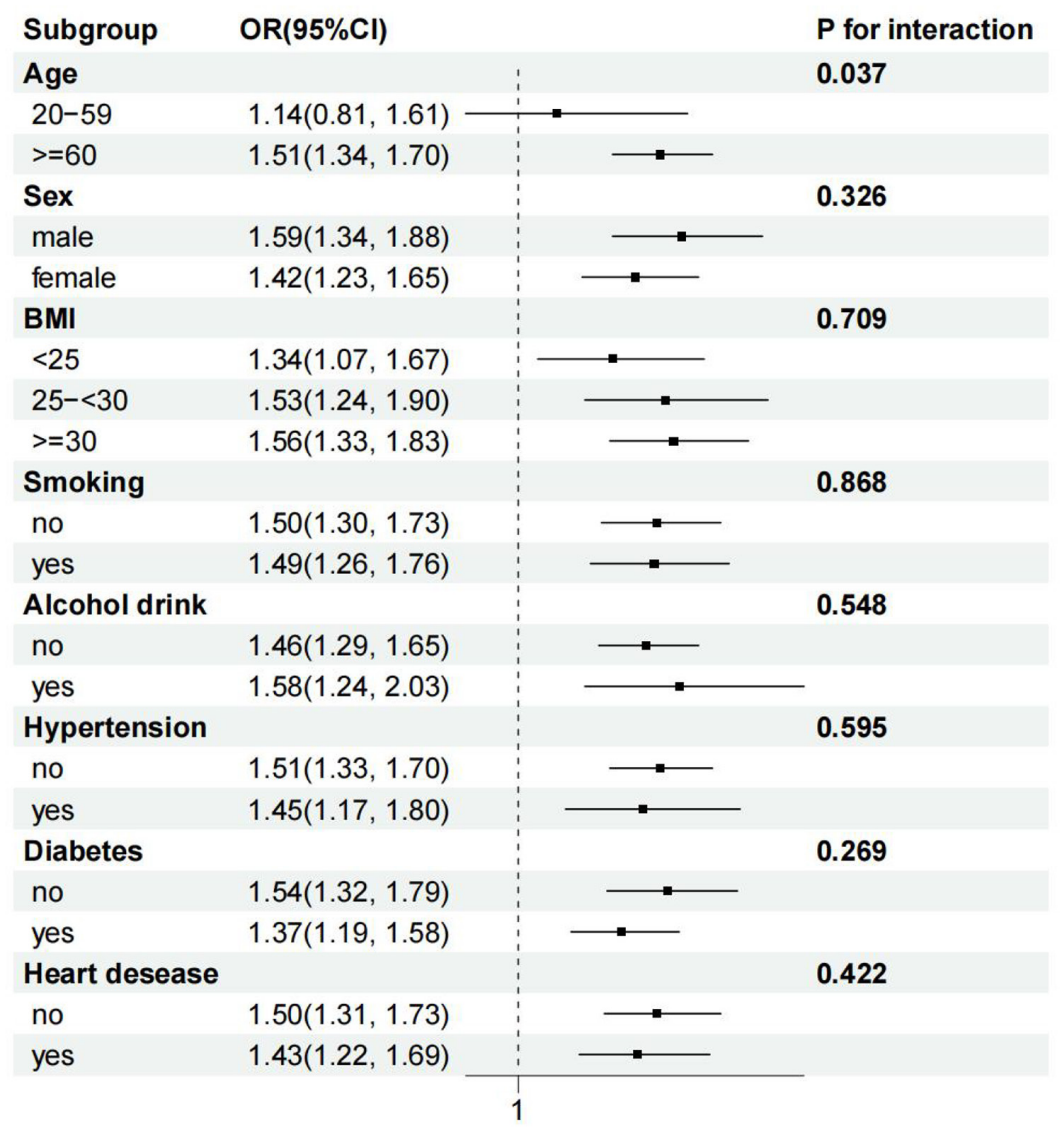
Subgroup analysis of the association between sleep duration and Alzheimer’s disease. Subgroup of age: 20 ≤ age ≤ 59; ≥60; subgroup of BMI: BMI < 25; 25 ≤ BMI < 30; BMI > =30. BMI = body mass index, CI = confidence interval, OR = odds ratio.

The logistic regression model based on AD showed associations between different sleep duration groups and AD. To assess the consistency of the sleep duration-AD association, we performed interaction analyses to investigate potential effect modifiers. We found that there was an interaction in the age subgroups (*P* = .037; Fig. 4); that is, this phenomenon could still be observed in people over 60 years old, whereas it was unstable in those under 60. The results of the subgroup analyses were stable.

## 4. Discussion

This cohort study investigated the association between sleep duration and AD in a representative US sample. Our comprehensive analysis of 29,692 participants revealed a significant positive correlation, wherein a longer sleep duration was associated with an increased incidence of AD. This association persisted even after extensive adjustment for sociodemographic factors, health behaviors, and comorbidities. Furthermore, analyses have confirmed that prolonged sleep is a positive promoter of all-cause mortality risk among patients with AD. Subgroup analyses demonstrated that the predictive validity of sleep duration for AD remained consistent across various strata, including smoking status, alcohol consumption, and chronic conditions such as hypertension and heart disease. Notably, both short and long sleep durations were positively correlated with AD prevalence, a pattern consistent with the known pathogenesis of AD in the elderly population. Collectively, these findings highlight the potential of sleep duration as a practical and effective predictor of AD.

Adequate sleep is fundamental to metabolic regulation. Substantial evidence has linked sleep disturbances to the pathogenesis of various conditions, including diabetes, obesity, and coronary heart disease,^[32]^ with insufficient sleep being recognized as a public health epidemic.^[33]^ Multiple determinants, spanning living environments, psychological factors, and comorbid diseases, modulate sleep patterns.^[34]^ In patients with dementia, prolonged sleep duration may stem from pathological alterations in brain regions governing sleep-wake cycles, such as the suprachiasmatic nucleus.^[35]^ Supporting this, studies have reported reduced levels of the wake-promoting neuropeptide hypocretin-1 and a corresponding loss of hypocretin-1 neurons in AD patients,^[36]^ potentially explaining the observed sleep extension. Given that the preclinical stage of dementia can extend over a decade,^[37]^ sustained long sleep duration may serve as a clinical predictor of an elevated AD risk. Although a 2-sample MR analysis did not establish causality, a significant association between long sleep duration and increased AD risk was consistently observed across all strata of the AD genetic risk score.

Accumulating evidence indicates that disruptions in the sleep-wake cycle, including insomnia, self-reported sleep disturbances, short sleep duration, fragmented sleep, and circadian rhythm abnormalities, are associated with an elevated risk of AD in humans.^[38-44]^ Concurrently, research in animal models has demonstrated that AD-promoting metabolites, Aβ and tau, accumulate in the brain,^[45-47]^ induce oxidative stress in the central nervous system, and compromise blood-brain barrier integrity,^[48]^ thereby potentially accelerating AD pathogenesis. This bidirectional relationship is further supported by the findings that AD pathology can disrupt sleep architecture.^[39]^ For instance, cerebral Aβ deposition has been shown to directly alter sleep patterns and circadian rhythms.^[49]^ Consequently, the assessment of sleep duration is critical for estimating AD risk, and our findings substantiate its utility as a practical indicator for the disease.

The integration of sleep duration metrics into practice by clinicians and social workers can significantly refine the accuracy of AD risk assessments. Social work interventions, including the establishment of robust support systems, delivery of health education, facilitation of access to medical resources, and provision of crisis management, constitute a comprehensive approach to proactive AD prevention. Individuals with prolonged sleep patterns benefit greatly from multifaceted support. This support not only reduces incidence and mortality rates but also enhances overall well-being. Therefore, social work plays an indispensable role in combating this disease.

Despite its contributions, this study had several limitations that warrant consideration. First, the cross-sectional design precludes causal inferences regarding the observed associations. Second, potential measurement inaccuracies in the collected covariates could have affected the precision of our estimates. Third, although statistically significant, the findings may lack immediate clinical translatability as the large sample size might detect minor differences that are not practically meaningful. Fourth, data constraints prevented the inclusion of potentially influential variables, such as sleep quality, circadian timing, and rhythm, which might have affected the observed relationships. Nonetheless, our study has notable strengths, including its foundation in a large, nationally representative NHANES dataset, which enhances the generalizability of our results. Furthermore, to our knowledge, this is the first study to specifically examine the link between sleep duration and AD while incorporating extensive subgroup analyses to test the robustness of the association.

## 5. Conclusions

In this cross-sectional study on AD, it was found that integrated sleep assessment can significantly improve the accuracy of AD risk prediction. This study highlights the clinical value of sleep duration as a predictive biomarker for AD and demonstrates its important role in disease risk assessment systems. Building on the predictive performance of sleep indicators, we further propose incorporating body composition parameters into AD risk stratification models to expand the scope of existing disease prevention strategies. These findings suggest that establishing a comprehensive evaluation framework that includes sleep monitoring and body composition analysis can provide new insights for early identification and precise intervention in high-risk AD populations. Future longitudinal studies across different populations are needed to validate the synergistic effects of multidimensional health indicators in the prevention and control of neurodegenerative diseases.

## Author contributions

**Conceptualization:** Yanli Liu.

**Formal analysis:** Guang Yang, Wenzhe Hou, Xian Yang, Ye Yang, Qiqi Chen.

**Methodology:** Guang Yang, Wenzhe Hou, Jiawen Wu, Xian Yang, Ye Yang, Qiqi Chen, Yanli Liu.

**Supervision:** Jiawen Wu, Yanli Liu.

**Writing – original draft:** Guang Yang, Wenzhe Hou, Xian Yang, Ye Yang, Qiqi Chen.

**Writing – review & editing:** Jiawen Wu, Yanli Liu.
